# Response to Letter to the Editor on “Development and validation of PEYRO-Q: a novel multidimensional patient-reported outcome measure for Peyronie’s disease”

**DOI:** 10.1093/sexmed/qfag081

**Published:** 2026-10-21

**Authors:** Andrea Cocci, Mattia Lo Re

**Affiliations:** Department of Experimental and Clinical Medicine, University of Florence, 50100 Florence, Italy; Department of Experimental and Clinical Medicine, University of Florence, 50100 Florence, Italy; Unit Urology and Andrology, University of Florence, Careggi Hospital, 50100 Florence, Italy

We thank the correspondents for their interest in the PEYRO-Q.^1^ We welcome scientific debate, but we must respond firmly: the letter mischaracterizes both the nature of our study and the standards of patient-reported outcome (PRO) measurement science, and its central recommendation—that the PEYRO-Q “should not be used”—is supported neither by the evidence we presented nor by the very methodological literature the correspondents cite.

The PEYRO-Q reported here is the *validated Italian version* of the instrument. Within its intended field of use—Italian-speaking clinical practice and observational research—it is a fully developed, content-valid, internally consistent, and temporally stable PRO measure, and it is fit for immediate use. The validation of any PRO is a cumulative, staged endeavor; the fact that the instrument’s scope will be broadened—to additional languages and to longitudinal responsiveness—does not render the demonstrated measurement properties provisional in any sense that would preclude its use for the purpose for which the instrument was built. We address the 5 points in turn; none of which withstands scrutiny as a reason to withhold the instrument.

## Cross-cultural validation

The correspondents fault us for not validating languages we never purported to validate. The PEYRO-Q presented here is explicitly the Italian version, supplied in full in its original language as Supplementary Material. A validated Italian instrument requires an Italian-language validation—which is precisely what we performed in a multicenter cohort with content validity established by a 22-member expert panel spanning 8 countries. Requiring French, German, or Spanish validation as a condition for using the Italian instrument in an Italian population inverts the logic of cross-cultural adaptation: adaptation extends an instrument’s reach; it is not a precondition for use in the source language. The International Society for Pharmacoeconomics and Outcomes Research (ISPOR) framework the correspondents invoke governs exactly the process we have scheduled for other languages—it does not retroactively invalidate a properly validated source-language instrument, and no measurement standard holds otherwise.^2^

## Factor structure and confirmatory factor analysis

The demand for a confirmatory factor analysis (CFA) is methodologically misplaced. Exploratory factor analysis is the correct and expected technique when a novel instrument’s dimensional structure is first examined; CFA tests a pre-specified model and must be conducted on an independent sample. Performing both on the same 206 respondents would be circular and is expressly discouraged by the COnsensus-based Standards for the selection of health status Measurement INstruments (COSMIN) guidance the correspondents themselves cite.^3^ Their objection therefore asks us to commit a methodological error we deliberately avoided—and to be criticized for it.

The further premise, that a “mismatch” between ten domains and 3 factors indicates poor construct validity, is incorrect. The 10 domains define the instrument’s content coverage; the 3 retained factors are higher-order latent dimensions of disease burden. Content structure and latent structure are distinct constructs and are not expected to coincide numerically. The coherent 3-factor solution, accounting for 62.2% of the variance and mapping cleanly onto clinical/functional burden, disease perception and evolution, and psychological/behavioral impact, supports—rather than undermines—the multidimensional design. The full rotated loading matrix is provided as Supplementary Material for readers who wish to verify the item-to-factor assignment directly.

## Concurrent/convergent validity

The assertion that the International Index of Erectile Function (IIEF) or Hospital Anxiety and Depression Scale (HADS) should have stood in for the PDQ misunderstands both our instrument and those comparators. The IIEF measures erectile function only; the HADS measures anxiety and depression only. Neither is a gold standard for the multidimensional, Peyronie’s-disease-specific burden the PEYRO-Q was built to quantify, and neither could serve as a criterion for the instrument as a whole. Convergent validity against such measures is meaningful only at the level of individual matched sub-domains and constitutes a refinement of an already content-valid instrument—not a precondition for its use. These targeted analyses form part of the program already under way.

## Statistic used for test–retest reliability

The claim of a “fundamental statistical error” rests on a misreading of Table 3. Test–retest reliability in our study was pre-specified and quantified by the weighted Cohen’s kappa coefficient (See “Methods”) and by the intraclass correlation coefficient (ICC), reported for the total score as 0.99 (95% CI, 0.98-0.99)—precisely the statistics the correspondents advocate. The per-item coefficients in Table 3 were computed across the 2 administrations of each item; for a variable measured on 2 occasions, this quantity is an agreement/reliability coefficient and is a special case of the ICC, not the item-homogeneity coefficient the correspondents describe. Our reliability conclusions therefore rest on the ICC and the weighted kappa, exactly as they should. We accept only that the column heading may state “ICC/agreement coefficient” more explicitly, and it will; the underlying analysis is correct and its conclusions are unchanged.^4^

## Responsiveness and minimum clinically important difference

Responsiveness and the minimum clinically important difference are, by definition, longitudinal properties. They cannot be—and never are—established in a development-and-reliability study, for the PEYRO-Q or for any PRO at first publication, including the PDQ the correspondents hold up as the benchmark. To demand them as a condition of use is to demand that no new instrument ever be used—which is not a tenable methodological position. For the cross-sectional and observational applications for which the PEYRO-Q is intended, responsiveness is not a prerequisite; for future longitudinal use it will be established through the distribution- and anchor-based methods we described in detail.

## Conclusion

In sum, the letter conflates the ordinary, staged extension of a validated instrument with a supposed lack of validity and elevates properties appropriate to later phases into preconditions for any use at all—a standard that no PRO in the field, the PDQ included, would meet at first publication. The PEYRO-Q is a content-valid, internally consistent, and temporally stable Italian PRO measure, ready for use in the clinical and observational settings for which it was designed. We are refining its presentation (explicit ICC labeling and the published loading matrix) and extending its scope (additional languages, convergent validity, and responsiveness) through the research program already in progress. We see no basis for the recommendation to withhold the instrument, and we are confident it will demonstrate its value in practice.

We thank the Editor for the opportunity to respond.
